# Implementation of distance learning IMCI training in rural districts of Tanzania

**DOI:** 10.1186/s12913-023-09061-y

**Published:** 2023-01-19

**Authors:** Kahabi Isangula, Esther Ngadaya, Alexander Manu, Mary Mmweteni, Doreen Philbert, Dorica Burengelo, Gibson Kagaruki, Mbazi Senkoro, Godfather Kimaro, Amos Kahwa, Fikiri Mazige, Felix Bundala, Nemes Iriya, Francis Donard, Caritas Kitinya, Victor Minja, Festo Nyakairo, Gagan Gupta, Luwei Pearson, Minjoon Kim, Sayoki Mfinanga, Ulrika Baker, Tedbabe Degefie Hailegebriel

**Affiliations:** 1 National Institute for Medical Research-Muhimbili Centre, Dar es Salaam, Tanzania; 2 Aga Khan University, Dar Es Salaam, Tanzania; 3 University of Ghana School of Public Health, Accra, Ghana; 4 London School of Hygiene and Tropical Medicine, Keppel Street, London, UK; 5 UNICEF Tanzania, Dar es Salaam, Tanzania; 6 Ministry of Health, Dodoma, Tanzania; 7 World Health Organization, Dar Es Salaam, Tanzania; 8 UNICEF Headquarters, New York, USA

**Keywords:** IMCI, PSBI, Tanzania, MCH, Newborn deaths

## Abstract

**Background:**

The standard face-to-face training for the integrated management of childhood illness (IMCI) continues to be plagued by concerns of low coverage of trainees, the prolonged absence of trainees from the health facility to attend training and the high cost of training. Consequently, the distance learning IMCI training model is increasingly being promoted to address some of these challenges in resource-limited settings. This paper examines participants’ accounts of the paper-based IMCI distance learning training programme in three district councils in Mbeya region, Tanzania.

**Methods:**

A cross-sectional qualitative descriptive design was employed as part of an endline evaluation study of the management of possible serious bacterial infection in Busokelo, Kyela and Mbarali district councils of Mbeya Region in Tanzania. Key informant interviews were conducted with purposefully selected policymakers, partners, programme managers and healthcare workers, including beneficiaries and training facilitators.

**Results:**

About 60 key informant interviews were conducted, of which 53% of participants were healthcare workers, including nurses, clinicians and pharmacists, and 22% were healthcare administrators, including district medical officers, reproductive and child health coordinators and programme officers. The findings indicate that the distance learning IMCI training model (DIMCI) was designed to address concerns about the standard IMCI model by enhancing efficiency, increasing outputs and reducing training costs. DIMCI included a mix of brief face-to-face orientation sessions, several weeks of self-directed learning, group discussions and brief face-to-face review sessions with facilitators. The DIMCI course covered topics related to management of sick newborns, referral decisions and reporting with nurses and clinicians as the main beneficiaries of the training. The problems with DIMCI included technological challenges related to limited access to proper learning technology (e.g., computers) and unfriendly learning materials. Personal challenges included work-study-family demands, and design and coordination challenges, including low financial incentives, which contributed to participants defaulting, and limited mentorship and follow-up due to limited funding and transport.

**Conclusion:**

DIMCI was implemented successfully in rural Tanzania. It facilitated the training of many healthcare workers at low cost and resulted in improved knowledge, competence and confidence among healthcare workers in managing sick newborns. However, technological, personal, and design and coordination challenges continue to face learners in rural areas; these will need to be addressed to maximize the success of DIMCI.

**Supplementary Information:**

The online version contains supplementary material available at 10.1186/s12913-023-09061-y.

## Introduction

Improving the capacity of healthcare workers (HCWs) to provide essential newborn care services has been widely recognized as a key entry point for reducing neonatal deaths. Cognizant of this, the World Health Organization (WHO), the United Nations Children’s Fund (UNICEF), and other partners have developed and supported the implementation of capacity building interventions for HCWs, including the integrated management of childhood illness (IMCI) strategy [1, 2]. Further, WHO has also developed a guideline on management of sick young infants with possible serious bacterial infection (PSBI) when a referral is not possible. The implementation of this guideline is documented as having the potential to contribute significantly to saving infant lives [3–6]. Improving the skills of HCWs on both IMCI and PSBI is a critical strategy for reducing neonatal deaths in resource-constrained settings [1–5]. This highlights the need for continued capacity building of HCWs in resource-limited settings to maximize their contribution towards preventing newborn deaths.

IMCI training focuses on improving case management among HCWs, health system strengthening and promotion of good practices at both the family and community levels. Evidence indicates that the IMCI strategy has the potential to both improve the quality of care and prevent neonatal mortality [7, 8]. Despite its potential, the standard face-to-face training model (a residential 11-day training) has faced several concerns, including poor trainee coverage efficiency; absenteeism of trainees from health facilities for prolonged periods of time, which negatively impacts service provision; and a high cost of implementation, especially in resource-limited settings [1, 2, 9]*.* Consequently, the distance learning IMCI training model (DIMCI) (10 weeks with only 3-day face-to-face meetings among trainees and facilitators) is increasingly being promoted to address some of these challenges.

More than 420,000 newborns die globally each year from serious infection. Most of these deaths could be averted by preventive measures, timely care seeking, treatment with appropriate antibiotics and follow-up [6]. Newborns in Tanzania are not exempted from developing signs of PSBI and requiring antibiotics. Evidence continues to indicate that infection-related neonatal deaths are a problem in Tanzania [10–15]. While the country has witnessed a rapid decline in under-five mortality, there has been a much slower decline in deaths of newborns in the first month of life [10]. The rate of mortality among infants (children aged below 12 months) is 43 per 1000 live births [10], while the neonatal mortality rate (deaths during the first 28 days) is 25 per 1000 live births and contributes to more than 50% of infant mortality. The major causes of newborn mortality in Tanzania include birth asphyxia (31%), complications of prematurity (25%) and infections (25%) [11], placing Tanzania among the top 10 countries with the highest number (thousands) of newborn deaths in the world, and among the top five in sub-Saharan Africa [12]. A survey by Mangu [13] indicates that sepsis contributed to 29% of 26,630 newborn deaths documented between 2005 and 2015, with an increase in hospital-based neonatal mortality rates from 2.6 deaths per 1000 live births in 2006 to 10.4 in 2015. This indicates that training of HCWs – to equip them with adequate knowledge and skills in identifying and timely managing sick newborns – is among the key strategies for reducing infection-related newborn deaths [6]. Cognizant of this, UNICEF supported delivery of the DIMCI training during implementation of a pilot project for the management of young infants with PSBI in Mbeya region of Tanzania for the past 3 years.

As the PSBI project is ending, it was important to conduct endline survey to examine how the DIMCI programme was implemented almost a decade after it started in Tanzania (See, [2]). This paper therefore examines participants’ accounts of the process used to implement the DIMCI training programme in Mbeya region. The paper draws from the data collected as part of an endline evaluation assessment that sought to assess and document the process used to implement the PSBI project, the outcomes achieved, and the lessons learned to inform recommendations for potential national scale-up based on the experiences of the three councils in Mbeya.

## Methods

### Design

A cross-sectional qualitative descriptive design was employed as part of an endline evaluation study of PSBI implementation in Mbeya Region of Tanzania. The endline evaluation took an implementation science approach, using mixed methods (qualitative and quantitative) for data collection. The use of qualitative descriptive approach for this inquiry was deemed appropriate to answer three key questions: (1) How was the DIMCI model implemented? (2) What were the achievements of the DIMCI model? and (3) what were the barriers encountered during implementation? A qualitative descriptive approach is appropriate for this inquiry as it aimed to develop an understanding and describe the implementation of DIMCI without testing an existing theory [16]. This approach offered an effective way of gaining a deep and rich understanding of the participants’ perceptions and experiences of DIMCI in the chosen context, as this may differ from other contexts in terms of culture, expectations and resources within health care settings. The qualitative data utilized for this paper were collected between June and August 2021.

### Settings

This endline study was conducted in three intervention districts – Busokelo, Kyela and Mbarali district councils – in Mbeya region. Mbeya region is one of Tanzania‘s 31 administrative regions and is located in the southwest part the country. The regional capital is the city of Mbeya. Mbeya Region is bordered to the northwest by Tabora region, to the northeast by Singida region, to the east by Iringa region, to the south by Songwe region and Malawi, and to the west by Songwe region. The region covers an area of 35,954 km^2^ and has a total of seven districts. There are total of 21 health facilities in Busokelo, 46 in Kyela and 56 in Mbarali district councils; all were included in the study. Each district has one district hospital. During the PSBI baseline study, the workforce density in Busokelo was 10 health professionals per 10,000 people. Nurses (registered, enrolled and midwives) were the most available cadre, while a limited number of specialists were found in the regional and referral hospitals in Mbeya.

### Sampling and participants enrollment

To document the process of implementation of the DIMCI training programme under the PSBI project, sampling commenced with a purposive selection of the three districts in Mbeya region, as well as national level officials and implementing partners. As noted above, all 21 health facilities in Busokelo, 46 in Kyela and 56 in Mbarali district councils were included in the study. We then conducted interviews with selective key informants, such as policymakers, partners, programme managers and HCWs (including beneficiaries and facilitators of the training programme). More specifically, interviews using the DIMCI key informant interview guide were conducted with purposefully selected national and district policy-level stakeholders and administrators (13) and HCWs working in child health outpatient clinics (32). This made for a total of 45 key informant interviews and in-depth interviews from the supply side. Contact information of national-level stakeholders was obtained from the newborn and child health unit of the Ministry of Health. Relatedly, contact information for Regional and district-level stakeholders, administrators and healthcare workers was obtained from the selected study sites’ regional and district medical offices. Then, courtesy calls were made with potential participants to inform them about the study and scheduling of the interviews considering the individual preference of time, date, and venue for those agreeing to participate. From the demand side, 15 in-depth interviews were conducted with purposefully selected community health workers (10) and exit interview clients (5), including mothers/fathers/caretakers with infants aged less than 60 days. Contact information of community health workers was obtained from the facility management team where DIMCI has been implemented. Courtesy calls were made to inform them about the study and scheduling of the interviews considering the individual preference of time, date, and venue for those agreeing to participate. Participants for exit interviews were recruited through reproductive and child healthcare clinics in the course of seeking routine care. Information about the study was communicated to mothers and caretakers of children with PBSI during the health education session. Those interested in participating expressed their readiness to the research assistant and were interviewed after receiving care but before leaving the facility.

### Data collection

Before data collection, the research assistants were trained on the use of data collection tools and techniques pertaining to this study. The English versions of the questionnaire were translated into Swahili language, then translated back to English and checked for conceptual equivalence. A consultative process was employed, involving experts at the National Institute for Medical Research and UNICEF, to generate and translate the interview guides into Swahili. Such tools were pre-tested in a purposefully selected setting. After pre-testing,data collection tools were refined to ensure that they were ready for use in the actual data collection process. Close and supportive supervision was done throughout data collection and analysis stages to ensure data quality. Both in-depth and key informant interviews were conducted in a quiet and isolated room entirely disconnected from regular activities. The audio-taped interview data were gathered using a flexible interview guide with topics on distance learning IMCI, including socio-demographic characteristics of the respondents, recruitment process, minimum qualifications, course content, structure and organization, learning and training approaches, mentorship and supervision, follow-up and course duration and schedule, job aids and beneficiaries. Perspectives of policymakers, partners, programme managers and HCWs (including beneficiaries and facilitators of the DIMCI training programme) were also examined. Prior to interviews, each participant was given an information sheet and a verbal description of the project in Swahili. Written or verbal consent was obtained or recorded respectively. Each key informant interview lasted approximately 60 minutes.

### Data management and analysis

During the training, project staff provided qualitative data collectors with digital recorders which they used to record, listen to and transcribe the interviews in Swahili. They then sent the transcriptions to translators, who translated the Swahili language into English in preparation for data analysis. Project staff saved the transcriptions – both the Swahili and English language versions – on password encrypted hard-drives or flash drives.

The initial analysis adopted a deductive ‘framework analysis’ approach for identifying themes and subthemes related to the research questions posed. The first stage of analysis involved reading through the transcripts to identify themes and subthemes and derive codes for subsequent stages of open and then axial coding. These steps broke down the qualitative data into units of analysis, and through this analysis researchers identified patterns and relationships, which elucidated the processes identified in the research questions. Explanatory matrices were then developed in relation to each theme, drawing on the patterns. The research team used a consensus-based approach to decide on including codes that did not fit within the pre-developed subthemes and themes; the codes were excluded when they did not provide critical value to the study, as confirmed by subjective and objective evaluations. This was followed by a collation of all relevant coded data extracts within identified themes. Peer consultation was ongoing throughout the analysis process, as the research team reflected on the codes and themes generated. Coded data within NVivo were then exported to Microsoft Word (Microsoft Corporation) for interpretative analysis and report generation. Participants’ accounts related to DIMCI were used for this paper.

As noted above, the research team did not use a theoretical framework; rather, participants’ descriptions of DIMCI were examined by considering training implementation in a specific context. This strategy allowed a contextualized exploration of issues related to implementation of DIMCI throughout data collection and analysis, without viewing it through an existing theoretical framework. Investigating DIMCI this way positioned our research within the constructivist paradigm, relying on participants’ descriptions to examine their perceptions and experiences of participation in the DIMCI in this specific context, rather than assuming it to be a positivist concept with a universally accepted framework of inquiry [17]. Future studies may consider a theory-driven inquiry in a similar context.

## Results

### Participants’ sociodemographic characteristics

This paper examines the data from qualitative interviews with 60 participants. Of the 60 participants, 53% were healthcare workers (nurses, clinicians and pharmacists); 22% were healthcare administrators (district medical officers, reproductive and child health coordinators and programme officers); 17% were community health workers; and 8% were mothers of young infants with PSBI.

### Key themes and subthemes

The results were heuristically grouped into three themes, namely: feasibility of DIMCI, efficacy of DIMCI, and cost efficiency of DIMCI. To consider feasibility, we examined issues related to course structure and organization, course beneficiaries, course contents, course delivery, mentorship, supportive supervision and follow-up, and availability of job aids. We then compared DIMCI with the standard IMCI training. To consider efficacy, we examined participants’ descriptions of the success of DIMCI implementation. Finally, to consider cost efficiency, we examined issues related to the cost of implementing the DIMCI programme. These issues are examined in detail in subsequent sections.

### Feasibility of DIMCI

Findings related to feasibility of DIMCI were sevenfold. The first issue related to feasibility of DIMCI from the implementation standpoint was course structure and organization. Project documents indicated that frontline health workers were trained through a 10-week distance learning course that consisted of three face-to-face meetings (facilitators and trainees) and two self-learning periods of 5 weeks each. The face-to-face meetings consisted of both classroom and clinical practice sessions in a nearby facility. During qualitative interviews, participants described the DIMCI course as including a brief face-to-face orientation session, several weeks of self-learning, group discussions within the facilities and/or neighbouring facilities, and brief face-to-face examination and review sessions. The complete training package was described as running for 10 weeks, with flexibility allowed in the schedule. The training schedule appears to have been pre-determined by the facilitators, while a consensus -building strategy was applied for the self-study and group discussion component after orientation. As a staff member from the Ministry of Health described:

*(DIMCI) runs for 10 weeks. [Participants] study for 5 weeks, come for the review session, then do five more weeks and come again and do the exam, which results in being awarded a certificate. There are three phases. They have 1 day for orientation, then they are given modules for self-reading. The orientation day includes the meaning of DIMCI and the guidelines they will use; they are given DVDs, guidelines and other materials covering several days [of the training]. Before they leave, they are put into study groups because there are days for self-reading and for group discussions. The group discussions have a chairperson. They develop a learning schedule and decide on topics for individual learning and group discussions. There are also groups for watching DVDs because we know some people residing in rural areas do not have TVs and some have no laptops, or don’t know how to use laptops. That is why they plan among themselves. (Ministry of Health staff member)*

The second issue related to feasibility of DIMCI was course content. The DIMCI training manual indicates the course contents as including: identification of signs such as fever, breathing rate, cough, diarrhoea, and ear problems; classification of severity of newborn based on their signs; management of identified newborn illness; education of mothers on home-based care and treatment; close follow-up of sick infants and documentation and reporting. During qualitative interviews, the contents of the DIMCI training were described as focusing on management of diseases of young children, including identification of danger signs, assessment and classification of severity using chartbooks, treatment and reporting. Specific newborn diseases covered included bacterial infections, diarrhoea and malaria. Decision-making was also covered, including referral decisions and initial management before referrals (e.g., dosage), as well as use of reporting tools and electronic system. Some participants commented:

*The IMCI training focused on diseases affecting young children, how to detect and investigate them, how to classify them as very severe, severe or not severe, and also treatment. We were also trained on how to use chart books for classification and filling the reports. (Trained HCW, Mbarali).*

*There were topics on classification of young children, topics on diarrhoea, malaria and other diseases affecting newborns, but also classifications of severity. There were also topics on management after classification, deciding on the need for referral, the services that must be offered before referral, and what to do if someone refuses referrals. They were therefore trained on complete management, dosage and duration, filling the tools and using electronic system in general. (Health administrator, Kyela).*

The third issue related to feasibility of DIMCI was the course beneficiaries. The project committed to training nurses and clinicians, specifically those working at the dispensaries and outpatient department of health centres and district hospitals through DIMCI, as they are directly- involved in newborn care in these facilities. Project documents indicated that a total of 430 health workers (covering 80% of eligible primary health care health workers) were trained from 174 health facilities (100% of health facilities in the project districts at the time of training). Qualitative interviews indicate that nurses and clinicians (clinical officers, assistant medical officers and medical doctors) were the main beneficiaries of the DIMCI training. The participant selection was described to be conducted by the district IMCI focal person following criteria set by the Ministry of Health. The selection criteria were based on cadres, primary responsibilities, level and ownership of facilities and areas with high numbers of young children. At least two participants were selected from dispensaries, including in-charges, nurses and in some, medical attendants. During qualitative interviews, the percentage of facilities in which HCWs participated in the training emerged as high, with some participants citing coverage ranging from 80% in Busokelo (reported by a trained HCW), 90% in Kyela (reported by a trained HCW) to 98% in Mbarali (reported by a health administrator). In Mbarali for example, 119 HCWs were cited as trained on DIMCI, although the target was 300. It is important to note that the reasons for not reaching the target were not clearly unpacked in qualitative interviews:

*We looked at cadres considering nurses and doctors according to the guideline, but most came from dispensaries because they are highly engaged in referrals. In all, we had 119 participants, although the target for the training was 300, because some faced different challenges that limited their participation. Therefore, we had facility in-charge nurses and other nurses, but there are some facilities in which medical attendants participated. We also had at least 1-2 participants from private facilities that offer outpatient care. We concentrated more on areas where we could get many young children. (Health administrator, Kyela).*

*Since the project primarily deals with reproductive issues, most of the participants selected are those who are engaged in reproductive issues, including nurses. Each facility produces about two participants working in reproductive health. We have 58 facilities, but two facilities are new. Therefore, about 98% of all facilities participated except new facilities, which had not been established when we developed the plans. (Health administrator, Mbarali).*

The fourth issue related to feasibility of DIMCI was course delivery. During qualitative interviews, as noted above, the delivery of DIMCI was described to include a mix of brief face-to-face orientation and review sessions, self-learning and group discussions. During brief face-to-face orientation and review sessions, DIMCI content was presented via presentations and demonstrations by facilitators, group discussions and assignments, and homework. Participants who described self-learning mentioned being given course modules, IMCI chart booklets, educative CDs/DVDs, IMCI photographic books, logbooks containing IMCI recording forms, and exam sheets. They further described meeting for group discussions, using the WhatsApp messaging platform for learning, as well as phone calls with facilitators for support when needed. Phone communication with facilitators was a concern because participants were responsible for the costs involved. This may have somewhat limited the frequency of calls, although such affirmations did not specifically emerge in the data. One participant commented:

*[Facilitators]were sending us photos of sick children recorded on CDs and we were also using books for reading and guiding treatments. To ensure that we were studying, they gave us exams that were collected and marked every time we met. Also, they gave us phone numbers for consultations whenever we faced any challenge, but we had to cover the cost of calling. There were study groups; each group had a leader and we used to agree on a meeting place for discussion on the cases using books and CDs. We used to communicate through SMS on where and when to meet, and the agenda and discussion questions. We used a computer to watch CDs (HCW, Mbarali).*

The fifth issue related to feasibility of DIMCI was mentorship, supportive supervision, and follow-up. Recognizing the importance of ongoing support after the training, the Ministry of Health and partners developed the ‘Guideline for follow-up after IMCI training’. This document provides guidance on key issues that need to be considered during follow-up after any IMCI training, with the purpose of reinforcing the new skills gained by participants and solving problems encountered in the course of implementing IMCI. A focus of mentorship and follow-up is, therefore, case management skills, health facility support (including availability of essential drugs and commodities for child health), and documentation and reporting of services offered. When asked about mentorship, supportive supervision, and follow-ups during the DIMCI programme, mixed descriptions emerged. Some participants described phone-based mentorship and follow-up by facilitators through WhatsApp and direct phone calls. Others mentioned brief face-to-face assessment and review assessment sessions after the participants had undertaken the assigned self-directed modules. In addition to assessments, the brief face-to-face review sessions with facilitators were cited as including discussion on challenges encountered during self-directed learning and clinical practice and distribution of additional modules. One participant commented:

*[Facilitators] came for follow-up after a certain time where they came to administer exams based on the modules we were given. For example, if we were given five modules, we were required to read them and answer the questions. When they came, they would ask for the assignments for marking and feedback. They asked about the challenges encountered on the modules and we discussed them together. Then they gave more modules for reading and responding to the questions, as well as the date for the next face-to-face session. (Health administrator, Busokelo).*

The sixth issue related to feasibility of DIMCI was availability of job aids. When asked about job aids, most participants cited learning materials such as modules, recording forms and DVDs. Likewise, child assessment and treatment decision-making materials, including IMCI chart books were noted. Additionally, reporting books or registers were reported to be offered by the project. While some participants described pre-existing working tools such as computers in their facilities, others cited logbooks and guidelines as the job aids provided. It is not clear whether participants were able to make full use of these materials; however, assessment of the logbooks during face-to-face review sessions was cited as an important monitoring strategy (see above).Regarding job aids, one participant commented:

*After finishing the training, I was given 13 books, including guidelines and logbooks. Each book described a certain disease, such as diarrhoea and others. We were required to read, answer questions, and fill out the logbooks for the module that we had completed. (Trained HCW, Mbarali).*

*They are given mother’s cards, charts books, 14 guidelines and DVDs. (Ministry of Health staff member).*

The problem with job aids, in particular the materials for self-learning DVDs, was inadequate facilities for viewing them, such as TVs and computers, concerns about the durability of DVDs (and consequently recommending that DVDs be converted into flash discs), and language barriers, with some recommending translation into Swahili for consumption even with low staff cadres. However, concerns about the cost of converting contents into flash disc were likewise highlighted as a potential limitation to this recommendation. Some participants commented:

*Some of us failed to answer the questions because they did not have facilities to watch the DVDs, and some encountered problems in using DVDs. (Trained HCW, Mbarali).*

*There were some challenges with DVDs. People living in rural areas take Bodaboda [motorcycle taxis] after class, which makes it easy for DVDs to scratch and they cannot be read afterwards [laughs]. Some do not have TVs or electricity, meaning they may need to watch from a neighbour’s home, after incurring the cost of fuel for generators. What they need to do is first convert them to flash discs and second translate DVDs into Swahili because they are in English, [a language] that people like medical attendants are not conversant in. (Ministry of Health staff member).*

The seventh and final issue related to feasibility was the comparison of DIMCI and the standard IMCI training. Qualitative interviews went further to explore participants’ perceptions about the differences between standard IMCI and DIMCI, with mixed views emerging. To better understand the comparison, we used heuristic criteria in describing the difference between DIMCI and standard face-to-face IMCI trainings. The first comparison criterion is the cost perspective. Participants were asked about their perceptions on the cost difference between the two models of IMCI training. Looking across transcripts, disagreements emerged regarding the cost of DIMCI compared with traditional face-to-face training. Most participants appeared to be unaware of the specific cost of DIMCI, but were able to offer comparisons. The majority of participants believed that the cost of DIMCI was less than the cost of traditional face-to-face IMCI trainings, as face-to-face trainings would require costly materials, such as venues, per diems, transport and prolonged engagement (i.e., number of days). DIMCI was therefore considered a cost-saving training approach by many participants, with one describing a cost reduction of 70 to 75%. Although both types of training require facilitation and training materials, the fewer number of days required for face-to-face orientation and the ability to train many participants at once were considered as the main cost-saving drivers of DIMCI:

*If we talk of resources, we consider cost reduction, which is 70% in distance learning, but other processes, including having a teacher come to teach, are almost the same. (Programme manager, Mbeya).*

*The cost per [DIMCI] participant is around US$340–400, but previously, [with IMCI] it used to be $1000. Therefore, the cost reduction is around 75% for DIMCI, including training materials plus follow-ups, but the (IMCI) used to be $1000 without follow-ups. (Manager, Mbeya).*

*Resources used for distance learning cost less than those for face-to-face training because when you invite people into a classroom you have to prepare notebooks, pens, pay for venue, food and transport. (Trained HCW, Busokelo).*

On the contrary, another group, comprised of a few participants, suggested that DIMCI may be expensive compared with face-to-face sessions. The need for face-to-face orientation and review sessions, materials for self-reading and the time-consuming nature of DIMCI were considered as the main drivers of cost compared with the standard IMCI. This suggests that for the cost of DIMCI to be less than that of standard IMCI, the training would need to be completely self-led, without any form of face-to-face interaction:

*I think distance learning may be expensive compared with face-to-face training because the latter runs and ends within a specific period. But distance learning involves meetings and being given assignments that you go and do. Then you come together to look at what you learned individually and you are given the next assignments. Therefore, it takes longer than face-to face learning. (Trained HCW, Mbarali).*

The second comparison criterion is beliefs on retaining the knowledge gained. Maximizing knowledge retention is a critical aspect of any training; if effective, it can reduce the need for frequent refresher trainings. Except for a few participants who considered the two approaches equally effective (i.e., having the same quality and knowledge decay potential), many considered standard face-to-face IMCI as having a higher likelihood of retention of the knowledge gained compared with DIMCI. The perceived drivers of high knowledge retention were an opportunity to gain more knowledge by prolonged interaction with facilitators conferred by standard IMCI compared with DIMCI. This was evident in the accounts of some trained HCWs who had first-hand experience with DIMCI:

*Learning through face-to-face has the potential to sustain knowledge for a long time. After all, a person studying face-to-face gains richer content and has more time to learn more things from facilitators, compared with distance learning. During face-to-face you interact and exchange ideas on many issues with facilitators, and you discuss with your fellows as well. The knowledge sticks in your mind for a very long time and you can use that knowledge to work effectively (Trained HCW, Busokelo).*

On the contrary, some macro-level participants suggested that DIMCI had more potential for knowledge retention than standard face-to-face IMCI. These assertions were very common in the accounts of WHO staff, who drew these conclusions from their rationale for developing the DIMCI programme or from their experiences in implementing the previous pilot project:

*After some time, skill retention was much better with DIMCI than with the standard IMCI because those in the standard scheme just went back after completing the course...but those in DIMCI had more time for self-reading and practice because they were more committed. (WHO staff member).*

The third comparison criterion is the work-study balance advantage. Since the trained HCWs are employed and working at healthcare facilities, a training that allows participants to study while continuing their regular work offers more work-study balance. Some participants affirmed that DIMCI provided more work-study flexibility compared with the standard face-to-face IMCI learning. Work-study flexibility was considered critical in maintaining the healthcare workforce at the facility, as it allows HCWs to fulfil their routine duties while studying. The standard IMCI was considered to offer less work-study balance because of the need for participants to attend trainings away from the workstation for a long time, creating a workforce deficit that negatively impaired service provision:

*Personally, I think DIMCI is good because a provider continues with normal work while studying. Assignments and scenarios will be sent, the provider reads the reference books, responds, and continues working. Many facilities will remain empty if the contents are delivered face-to-face and providers must attend (face-to-face) trainings, because the topics are very long, and they study for a very long time. Therefore, it will contribute to a staffing deficit at the facility and impair services. DIMCI is good because they study and continue offering services. (Health administrator, Busokelo).*

*[DIMCI came because] people were complaining about the problem with face-to face training* – *that it requires taking a provider away from a workstation for almost 2 weeks, meaning people were missing the services. That is why it was necessary to come up with a modality in which the provider is taken away for a very short time, but receives the same content as if she or he were taken for a long time. (National trainer of trainers).*

The fourth comparison criterion is the potential to allow reflective critical thinking. Aside from one participant, who considered both DIMCI and face-to-face trainings as having the same quality, some felt that DIMCI provided more opportunities for reflective critical thinking on the content than standard face-to-face IMCI training. Self-learning in DIMCI was said to allow for self-reflection among participants, while the standard face-to-face IMCI was considered to be a form of ‘spoon feeding’ the contents. Some went further to suggest that DIMCI increased the motivation for self-directed study compared with standard face-to-face IMCI:

*Distance learning facilitates learners’ capacity to think more critically and expand their thinking on their own, compared with trainings where you are taught everything. Because in distance learning, imagine you meet a case, you must discuss among yourselves as providers, challenge and correct one another until you reach current management*. *[This is] unlike ‘spoon feeding’ in a classroom, [where] everything is taught by a trainer. (Health Administrator, Kyela).*

*IMCI did not build the culture of self-study and people had lost motivation to study, but DIMCI built a studying culture. You may find a facility has three to four staff and they can study together and motivate each other. DIMCI has facilitated easy implementation because learning occurs at the facility; therefore, a HCWs implements everything she or he studies at the same facility. (WHO, staff member).*

The fifth and final comparison criteria is the number of beneficiaries. There was broad consensus among participants that DIMCI offered an opportunity for more people to learn at the same time, compared to the standard face-to-face IMCI training. This indicates that DIMCI has the potential to reach more people than the standard IMCI, especially if no face-to-face orientation sessions are included. Furthermore, DIMCI was considered to offer more opportunity for skills practice because participants had greater access to sick newborns at their workstation during self-directed learning, compared with limited interaction with cases during standard face-to-face trainings:

*Distance learning is very good because many people get educated at the same time, instead of taking one person from the facility to go attend the training for seven or 14 days. (HCW, Mbarali).*

*During standard IMCI, it was difficult to get sick newborns for practice; therefore, we ended with just demonstrations. But with DIMCI, they can access sick newborns at the facility every day and they were able to go to a nearby facility or visit them at home and they had more time to do assessments of young children. (WHO staff member).*

### Efficacy of DIMCI implementation

Participants cited several successes arising from the application of the skills they had gained through the DIMCI training programme, including: (i) improved knowledge among HCWs on IMCI; (ii) improved management of under-five children due to improved knowledge and skills; (iii) improved quality of care and; (iii) improved happiness among providers and service users. According to participants, increased happiness among service users was largely influenced by reduced waiting time and improved friendliness of healthcare providers (detailed previously). Such improvements were highly linked to the DIMCI training; they not only contributed to reducing newborn and maternal deaths, but will likely continue reducing deaths of newborns and mothers in the future:

*Providers became very happy. Service users became very happy. Services were accessible. Community health workers were available and working responsibly. We have reduced deaths to a large extent. (UNICEF staff, Mbeya).*

*This project is very good. The project has brought many successes, especially in offering care to under-five children. Most providers did not have a good understanding of the management of young children, but this has improved after the distance learning, especially after learning through modules and practising afterwards. They use chart books; they have the capacity to refer and know what is needed. I believe in the next 10 years, deaths among under-fives will be reduced significantly, because even now, you can’t compare with what was happening before. (Health administrator, Mbarali).*

Another success cited by participants was improved confidence and capacity to identify and manage problems experienced by young children through a classification process using chartbooks. This was the dominant success cited in relation to the knowledge and skills gained through the DIMCI training. Increased confidence in managing newborn diseases among healthcare workers was likewise linked to reduced newborn referral tendencies from low to higher level facilities compared with the pre-project period. Furthermore, there was an affirmation of improved use of the IMCI guideline for management of childhood diseases. Increased use of the IMCI guideline among healthcare workers for treatment decisions may have contributed to improved management of newborn diseases:

*One of the important benefits is increased confidence of healthcare workers in managing newborns. Before that, HCWs at lower levels were just referring newborns, even if they had the capacity to manage. Therefore, DIMCI built the capacity of HCWs to manage young children at low levels. (WHO staff member).*

*Personally, the successes I have witnessed include children who met the criteria for severe diseases after using classification procedures. This has simplified our work because we do not need to use complex investigations to detect problems that a child is suffering from. You just open your chart book and classify the baby based on the symptoms described by the mother. This has made it easy to discover the problems troubling the child and offer effective treatment as part of IMCI. (Trained HCW, Mbarali).*

*The training was good. Initially, I did not know how to recognize a child with pneumonia but now I can detect him/her. I can treat the baby (with pneumonia) very well without any problem. In short, the training has helped us a lot; for example, if the doctor is not available, I can sit and treat a baby without any problem. (HCW, Mbarali).*

### The cost efficiency of the DIMCI programme

Qualitative interviews went further to examine training costs. Looking across transcripts, there was broad consensus among participants that they were unaware of the cost of DIMCI trainings. Most participants, such as trained HCWs and district administrators, such as district medical officers and district RCH cordinators used phrases such as, ‘I cannot talk about it’ or ‘I don’t know the cost incurred’. Such statements suggest that the DIMCI budget may not have been shared with district authorities and participants. However, few health administrators went ahead to mention cost items, such as per diems paid during a brief face-to-face DIMCI orientation training (TZS 80,000 for each participant from the district). Other costs included transport refunds, food, venue and stationery, particularly during the brief face-to-face sessions within DIMCI training. One health administrator approximated the cost of DIMCI to reach about TZS 27 million per session:

*I cannot talk about the cost of training because I do not know how much the [PSBI] implementers used, but on our side, we paid a large cost, especially during participant meetings with facilitators. (Health administrator, Kyela).*

*The cost per participant for the per diem at the district was TZS 80,000. Transport was also refunded at about TZS 10,000 each. I did not capture the full cost because the activity was coordinated by Catholic Relief Services and UNICEF. But there were also food costs; I don’t know how much they paid. There was also stationery, including notebook and printed papers. Also, they hired three venues, so almost TZS 14 million and TZS 40,000 may have been used per participant. We had seven facilitators; I don’t know how much they were paid, but let us say they got TZS 150,000 … they may have used TZS 9 million each, making a total of around 27 million (Health administrator, Mbarali).*

## Discussion

This paper describes the delivery of the DIMCI training programme during PSBI project implementation in the three district councils in Mbeya region. We utilized the data collected as part of an end-line evaluation that sought to assess and document the process used to implement the PSBI project, the outcomes achieved, and the lessons learned in the three councils to inform recommendations for potential national scale-up. Qualitative interviews were conducted with national, regional and district stakeholders, including trained beneficiaries and implementing partners, to generate an understanding of how distance IMCI was implemented. The study was constructed with the acknowledgement that prompt identification and treatment of sick young infants (aged 0 to 59 days) is key in reducing mortality and morbidity [3–5]. While several interventions exist for the care of sick newborns in healthcare facilities, newborn morbidity and mortality remain challenges in Tanzania. As part of the response, UNICEF supported the Government of Tanzania to implement a three-year pilot project in the Mbeya region applying the new WHO PSBI guidelines in primary health facilities to provide guidance on the use of simplified antibiotic regimens. As part of PSBI implementation, HCWs were trained using the DIMCI training curriculum. The pilot project was constructed within the context of scientific evidence to the effect that implementation of the WHO guideline on the management of sick young infants with PSBI when a referral is not possible can contribute significantly to saving infant lives [3–5]. Assessing the impact of the intervention, documenting the process of implementation of the distance learning IMCI programme and documenting the key lessons learned was therefore critical in offering recommendations that could inform scale-up both within and outside the country.

### The origin of DIMCI in Tanzania

Examining its origin in Tanzania, the findings of the present study indicate that DIMCI originated from standard IMCI because of the need to enhance efficiency and deliver more cost-effective training courses. This need was fuelled by the desire to reduce training costs by reducing the number of days required for face-to-face training, maximizing the number of participants using limited resources, and reducing the prolonged absence of HCWs at facilities during class-based IMCI trainings (leading to service delays). Low coverage of IMCI, its high cost and the need for HCWs to be away from their workplace for a prolonged period, have been previously documented as common shortfalls of the standard face-to-face IMCI training [1, 2, 9]. Distance learning IMCI has been considered an innovative and low-cost alternative for addressing these gaps in the standard face-to-face IMCI training [2]. This implies that delivering IMCI through a distance learning model could be an important strategy for building the capacity of the healthcare workforce without requiring travel away from the workstation. These findings further indicate that DIMCI was also influenced by the global movements on PSBI, including global meetings for dissemination of recommendations for management of newborns. Consequently, local guidelines were reviewed, training modules and chartbooks were developed, and facilitators were trained with a pilot in the three districts. This may explain why the DIMCI programme implemented in Mbeya was adapted from WHO distance learning IMCI training curriculum [18]. This further implies that the DIMCI was implemented in Tanzania by adhering to global recommendations on implementing similar activities in low-resource countries. 

### The delivery of the DIMCI: Structure, organization, contents, and beneficiaries

Our findings indicate that the delivery of DIMCI involved a face-to-face orientation session, several weeks of self-learning, group discussions involving healthcare workers within facilities and/or neighbouring facilities, and brief face-to-face examination and review sessions. The course contents included topics such as identification of danger signs, assessment and classification of severity using chartbooks, referral decisions and initial management before referrals (e.g., dosage), and the use of reporting tools and electronic system. Therefore, a focus on topics reflecting the major conditions contributing to newborn and under-five mortality in Tanzania was critical for maximizing the benefits of DIMCI. It is important to note that infections, delayed treatment, and delayed referrals have been previously documented as among the key contributors of newborn and under-five deaths in Tanzania, despite notable improvements [13, 19, 20]. Furthermore, research continues to indicate that frontline health workers have weaker capacity to provide quality and timely maternal and newborn care in Tanzania, with most needing additional training [21]. Therefore, a focus on nurses, clinical officers, assistant medical officers, and medical doctors is a critical aspect of DIMCI because they are the first individuals to handle sick newborns in primary healthcare settings. The selection of these frontline healthcare workers was conducted by a district IMCI focal person in coordination with the reproductive and child health coordinator and the Ministry of Health, partly because these people understand the capacity gaps within the healthcare system. Taken together, these findings imply that despite being delivered using a distance model, DIMCI was packed with topics that aimed to enhance the capacity of frontline healthcare workers to detect and manage newborns with PSBI in an attempt to increase their survival.

The findings indicate that during the brief face-to-face orientation, DIMCI content was delivered via presentations and demonstrations by facilitators, group discussions and assignments, and homework. Self-directed learning was delivered via course modules, IMCI chart booklets, educative CDs/DVDs, photographic books, logbooks and exam sheets. Our findings largely reflect what has been documented in previous literature on implementation of DIMCI in Tanzania [2, 21–23]. Muhe [2], for instance, documented DIMCI as consisting of three face-to-face encounters between IMCI trainees and IMCI facilitators and two self-study periods (3–4 weeks and 8–9 weeks) with self-directed learning for 10–12 weeks for 4806 healthcare providers trained in 68 districts in Tanzania. This indicates that, in low resources settings, brief orientation and follow-up sessions are often needed on top of the self-directed learning, which may pose significant costs for the delivery of DIMCI.

### The comparison between DIMCI and the standard IMCI

A comparison of DIMCI and the standard face-to-face IMCI training model was carried out. Similar to the standard IMCI, DIMCI is expected to include mentorship and follow-up activities as part of continued support for learners. Follow-up visits are expected to be conducted 4–6 weeks after training to assess clinical skills, reinforce clinical skills as well as provide supportive supervision, solve supply issues and ensure reporting [2]. However, our findings indicate some weakness in mentorship and follow-up of HCWs after the DIMCI training, with reliance on phone-based consultations with facilitators and peer group discussions. It is important to note that lack of mentoring and supervision from the tertiary level has been documented as one of the key barriers to implementation of IMCI among HCWs in Tanzania [23]. While poor mentorship and follow-up after DIMCI training may be partly explained by inadequate funding and transportation after funding has ceased, this may have contributed to a preference for standard IMCI training among some participants. This suggests a need for strengthening facility-based mentorship, supportive supervision and follow-up activities during and after DIMCI training. The successful implementation of DIMCI may require well-structured mentorship and follow-up activities. As such, the budget for supportive supervision may need to be increased for subsequent DIMCI implementation.

### The challenges of DIMCI implementation

Our findings indicate that the problems encountered during DIMCI implementation included technological issues, such as inadequate facilities for personalized learning (e.g., TVs and computers) and the non-durability of DVDs. Technological challenges have been previously indicated as limiting the capacity of both HCWs and medical students to fully utilize the benefits of distance learning courses in Africa [24–26]. This suggests a need to ensure access to relevant technology among learners and the need for DIMCI materials to be available in multiple formats (e.g., DVDs and flash discs) to accommodate people who are unable to make use of the materials. The second challenge was personal issues, such as limited time for self-study due to competing work and family responsibilities and language barriers (with some recommending translation of contents into Kiswahili). Competing priorities among HCWs has been documented as a key challenge of implementing IMCI in Tanzania [23]. Nevertheless, language barriers suggest the need for translation of DIMCI materials into Swahili to ensure effective content delivery and absorption by HCWs within the country. The final challenge was design and coordination issues, such as low financial incentives and inadequate funds for mentorship, supervision and follow-up. These challenges may explain why there were mixed preferences for standard and distance IMCI, with some people expressing preference for distance IMCI because of its relatively lower cost and its ability to offer better work-study balance and critical thinking, while others preferred the standard IMCI because of the high possibility of knowledge retention. Most of these issues have been documented as common in other distance learning training models focusing on HCWs in Tanzania and other low-income settings [27–29]. Taken together, these findings indicate that, although DIMCI may be less expensive than standard IMCI, there is a need to address the challenges of DIMCI by considering the technological, personal and coordination barriers that HCWs in rural areas continue to face to maximize its success.

### The success of DIMCI implementation

Despite the challenges observed, the findings indicate that DIMCI successfully facilitated the training of many healthcare workers, without jeopardizing patient management and at a low cost. DIMCI was linked to improved knowledge among HCWs, and improved competence in the management of under-five children. Such improvement was regarded as more likely to reduce deaths of newborns and mothers in future, with some participants affirming that the training had contributed to a reduction in newborn deaths. Other successes included improved confidence and capacity to identify and manage problems suffered by young children through the classification process using chartbooks, and improved use of the IMCI guideline for the management of childhood diseases. Similar findings have been reported in previous studies. For instance, Muhe [2] reported that DIMCI allowed many HCWs to be trained in parallel and that HCWs trained in DIMCI performed equally well as those trained in the standard IMCI. These findings need to be considered with caution as increased confidence and competence noted may deter HCWs at low-level facilities from providing timely referrals to some young infants with PSBI.

## Conclusion

The DIMCI appears to have been implemented successfully in rural Tanzania. DIMCI facilitated the training of many HCWs at a low cost and resulted into improved knowledge, competence and confidence among HCWs in the management of sick newborns. However, technological challenges related to limited access to proper learning technology and language barriers for IMCI, personal challenges including work-study-family demands, and DIMCI design and coordination challenges, including low financial incentives and limited subsequent mentorship and follow-up, continue to face learners in rural areas. These challenges will need to be addressed to maximize the success of DIMCI.

## Supplementary Information

**Additional file 1.**

## Data Availability

The datasets used and/or analysed during the current study are available from the corresponding author on reasonable request.

## Abbreviations

CD: Compact disc
DIMCI: Distance learning on the integrated management of childhood illness
DMO: District medical officer
DVD: Digital versatile disc
HCW: Healthcare worker
IMCI: Integrated management of childhood illness
NIMR: National Institute for Medical Research
PSBI: Possible serious bacterial infections
UNICEF: United Nations Children’s Fund
WHO: World Health Organization
