# Supplementary material for: Implementation of distance learning IMCI training in rural districts of Tanzania
Source: BMC Health Serv Res. 2023 Jan 19;23:56. doi: 10.1186/s12913-023-09061-y (PMC9854197; doi:10.1186/s12913-023-09061-y)
Supplement: Supplementary file 1 — Additional file 1. [file 12913_2023_9061_MOESM1_ESM.docx]

**INTERVIEW GUIDE**

**(Qualitative KII)**

ID of interviewee……………………………………………………… … Sex……………….

Designation…………………………………................... Place…………………………………..

Marital status ( ) married ( ) single ( ) divorced ( ) Separated ( ) widow

Date of Interview ……………………………………………

Highest level of education:

Duration in years for the current post (designation): ……………………..Years

**Questions:** *(Answers must be audio-recorded)*

1. What roles did you perform in implementation of DIMCI??
2. What were the target participants for DIMCI? (Probe: *minimum qualifications, recruitment process)*
3. What were the DIMCI course contents? (Probe: *Topics covered, structure, schedule, duration and organization)*
4. How was the DIMCI course delivered? (Probe*: training modality, learning approaches, learning resources, course duration,)*
5. How was follow-up, mentorship and supervision performed? *Probe: frequency, support provided*
6. What job aids were provided after DIMCI training?
7. How DIMCI differs from Standard IMCI training? (Probe: *contents, delivery modality, job aids and support, cost etc)*
8. Any other comment?

Name of interviewer ………………………………………………………………………………
